# Meteorology—Mason’s Hygrometer

**Published:** 1855-09

**Authors:** James A. Kirkpatrick

**Affiliations:** Central High School, Philadelphia


### *Meteorology—Mason's Hygrometer.*

By James A. Kirkpatrick, A. M.

The *Force of Vapor*, *Relative Humidity*, and *Dew Point*, may all be calculated from the indications of Mason's Hygrometer. This simple and convenient instrument consists of two thermometers whose indications agree perfectly, when they are both dry. One of the thermometer bulbs, however, is kept wet, by a piece of thin muslin, the end of which dips into a fountain of water, similar in construction to the fountains attached to bird-cages. This permanent fountain constitutes the principal difference between Mason's Hygrometer and August's Psychrometer. The water evaporating from the moistened bulb, lowers its temperature, in proportion to the rapidity of its evaporation, and thus gives the means of obtaining the most important results.

From experiments carefully made, it has long been ascertained that the vapor of water, filling any space to saturation, exerts a certain expansive force, pressure, or tension, which varies with the temperature of the air. This is called the Maximum Tension or Elastic Force of Vapor of Water, for that particular temperature. For instance, it has been found that for 3°.2, F., the Maximum Tension or Force of Vapor is equal to $\frac{1}{20}$ of an inch of mercury; for 18°. 3, to $\frac{1}{10}$ of an inch; for 32° to $\frac{18}{100}$ of an inch; for $40\frac{1}{4}$° to $\frac{1}{4}$ of an inch; for 59° to $\frac{1}{2}$ an inch; for 79°.3 to 1 inch; and for 101°.4 to 2 inches of mercury, &c. From these experiments a table of the Maximum Tension, or Elastic Force of Vapor, has been constructed by Regnault, and published by the Smithsonian Institution, but in French measures,—that is, the temperature is expressed in Centigrade degrees, and the height of the mercurial column in millimetres. I find, however, that Prof. Boye in his small but valuable treatise on Pneumatics, lately published, has converted these French measures into English inches and Fahrenheit degrees. With the use of this table, and the following formulæ, the Force of Vapor existing in the atmosphere, the Relative Humidity and the Dew Point, quantities always interesting to the meteorologist, may be easily and accurately calculated.

Let $t$, represent the temperature of the air in Fahr. degrees, as indicated by the dry-bulb thermometer.

$t'$, the temperature indicated by the wet-bulb thermometer.

$f$, the Force of Vapor in the atmosphere at the time of observation;

$f'$, the Maximum Tension in inches of mercury, for the temperature ($t'$) of the wet-bulb thermometer, as exhibited in the table;

$f''$, the Maximum Tension for the temperature ($t$) of the dry-bulb thermometer;

$d$, the Dew Point, at the time of observation;

$h$, the Relative Humidity; and

$b$, the height of the mercury in the Barometer in English inches. Then

*To find the Force of Vapor.*

$$f = f' - \frac{0.480(t-t')}{1130-t'} b.$$

If, however, the temperature is below the freezing point, and the wet bulb is covered with a film of ice the formula becomes

$$f = f' - \frac{0.480\,(t-t')}{1272.2-t'} b.$$

By *Relative Humidity* we mean the proportion which the Vapor of Water, existing in the atmosphere, bears to the maximum quantity, or the quantity necessary for saturation at the existing temperature. That is, a Relative Humidity of .55, means, that the atmosphere contains $\frac{55}{100}$ of the quantity of vapor which, at the existing temperature, is necessary to saturation.

*To find the Relative Humidity.*

$$h = \frac{f}{f''}.$$

*To find the Dew Point.*—As the force of vapor in the atmosphere, at the existing temperature, must be the same as the maximum force corresponding to its dew point, or *the tempera-*

*ture at which vapors begin to condense like dew, on any cold surface,* it follows that the degrees of temperature in the table of Maximum Tension corresponding to the force of vapor ($f$) in the atmosphere, will be the Dew Point. It is in this way that the Dew Point has been calculated for the Medical Examiner for the last two months.

To illustrate these formulæ, let the dry bulb be 85°, the wet bulb 74°, and the barometric pressure 30.0 inches; then

$$f = 0.8390 - \left(\frac{0.480 \times 11}{1130 - 74}\right) \times 30.0 = 0.839 - 0.150 = 0.689 =$$

force of Vapor in the atmosphere.

$$h = \frac{f}{f''} = \frac{0.689}{1.2030} = 0.57 = \text{Relative Humidity.}$$

Then $d$ = the temperature corresponding to $f$ in the table which is found by inspection to be 68°.2.

Considerable care is necessary in the use of this Hygrometer. It should be fixed permanently in the shade, in the open air, protected from rain, and from reflected heat. When the temperature is near the freezing point, some difficulty arises from the uncertainty in the freezing of the water on the bulb, but this seldom happens, and may be remedied by more careful observation. The water with which the fountain is filled, should be pure rain water, or water that has been boiled. The muslin covering the wet bulb should be cleaned whenever any impurities appear upon it, and renewed at least four times a year.

Central High School, Philadelphia, July, 1855.

